# Low-Grade Gastric Fibromyxoid Sarcoma in an Adult Female: A Rare Case Report

**DOI:** 10.1055/s-0046-1820425

**Published:** 2026-05-11

**Authors:** Duaa Al-shurbaji, Muna AL-Khawaldeh, Layth H. Zraygat, Mohammad Al-Slehat, Rula Al-Shimi

**Affiliations:** 1 Department of Histopathology, King Hussein Medical Center, Jordanian Royal Medical Services, Amman, Jordan; 2 Department of Oncology, King Hussein Medical Center, Jordanian Royal Medical Services, Amman, Jordan; 3 Department of Medicine, Jordan University Hospital, Amman, Jordan; 4 Faculty of Medicine, Jordan University of Science and Technology, Irbid, Jordan

**Keywords:** low-grade fibromyxoid sarcoma, gastric, FUS-CREB3L1 fusion

## Abstract

Low-grade fibromyxoid sarcoma (LGFMS) is an uncommon soft tissue tumor that often masquerades as benign due to its deceptively mild histopathological appearance. Predominantly arising from the deep soft tissues of the extremities and trunk, LGFMS is exceptionally uncommon in the gastrointestinal (GI) tract. While most documented cases have been found in the small intestine and colon, only one prior case has been reported in the stomach involving an elderly Japanese woman. In this report, we present a compelling case of gastric LGFMS in a 21-year-old female who experienced mild hematemesis and fatigue. An abdominal computed tomography scan unveiled an ill-defined, hypodense mass originating from the lesser curvature of the stomach and extending toward the left hepatic lobe—highlighting the complexity of this condition. Further investigation included an upper endoscopy and an incomplete laparoscopic resection of the tumor. Histopathological analysis revealed a proliferation of spindle cells with focal whorling within a heavily collagenized stroma, transitioning abruptly to a myxoid area, thus confirming a diagnosis of LGFMS. Immunohistochemical testing showed positive results for vimentin and BCL2. Crucially, molecular analysis identified the FUS-CREB3L1 fusion. Based on our search in the literature review, this case is considered a second instance reported in English literature.

## Introduction

Low-grade fibromyxoid sarcoma (LGFMS) is a rare subtype of fibrosarcoma first identified by Evans in 1987. 1 It is most frequently present in the soft tissues embedded in the central body region and lower limbs, mainly affecting young to middle-aged groups. 2 However, despite its rarity, cases have been documented in less uncommon sites such as the retroperitoneum, small intestine, and colon. 3 4 5

Histologically, LGFMS demonstrates a benign morphological appearance and originates from deep soft tissue. 6 Despite its indolent histopathological features, the tumor tends to have distant metastasis and local recurrence. 7 Molecular analysis using reverse transcription-polymerase chain reaction (RT-PCR) has demonstrated the presence of the FUS-CREB3L2 fusion gene in the majority of neoplastic cells, serving as a crucial diagnostic indicator for LGFMS. 8

Here, we presented a gastric (LGFMS) case in a young female, highlighting an unusual anatomical site of this rare malignancy. Based on our literature review, this case represents a second gastric LGFMS.

## Case Presentation

A 21-year-old female presented to the surgery clinic at the Jordanian Royal Medical Services (JRMS) with mild hematemesis and fatigue. She had a history of recurrent unexplained anemia 1 year prior.

An abdominal computed tomography (CT) scan, performed without contrast, revealed a gastric mass. As a result, she was referred to the Military Oncology Center for further evaluation and management.

**Fig. 1:**
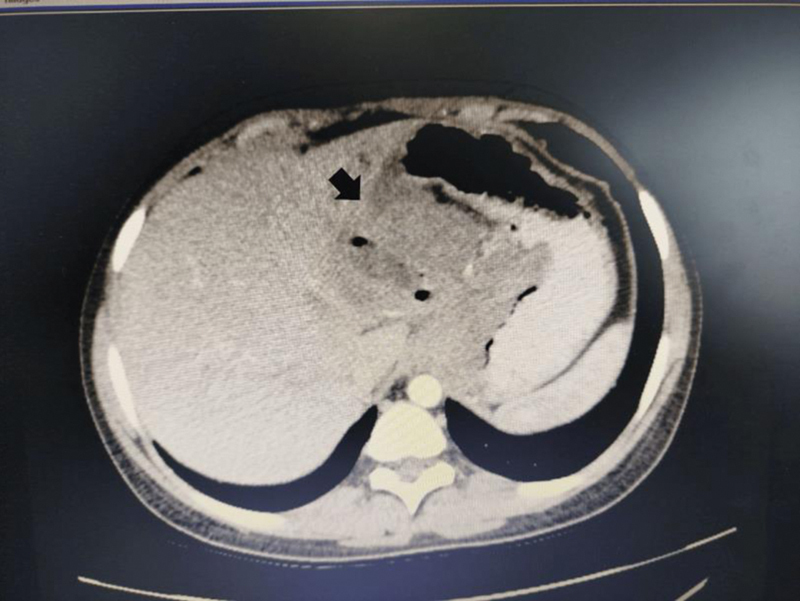
Axial CT scan showing multiple hypodense liver lesions ( *arrow* ).

Subsequent CT imaging with contrast showed a large, lobulated, ill-defined, hypodense heterogeneous mass arising from the lesser curvature of the stomach and extending to the left lobe of the liver, as illustrated in Fig. 1

Further lesion characterization was conducted using magnetic resonance imaging (MRI). The axial view of the T2 sequence revealed a significant lobulated soft tissue mass of varying attenuation, originating from the stomach and extending into the left lobe of the liver. These findings confirmed that the lesion originated in the stomach and exhibited extension toward the left hepatic lobe.

An upper endoscopy was performed to evaluate the patient's lesion. The examination identified a large polypoidal mass in the proximal stomach that extended into the distal esophagus, resulting in luminal obstruction. The mass was predominantly situated along the lesser curvature and involved the gastric cardia. Endoscopic assessment revealed ulceration and necrotic tissue on the lesion's surface, and multiple endoscopic biopsies were taken from the gastric lesion.

The microscopic examination of the tissue sample for histological evaluation, was stained with hematoxylin and eosin (H&E).

**Fig. 2:**
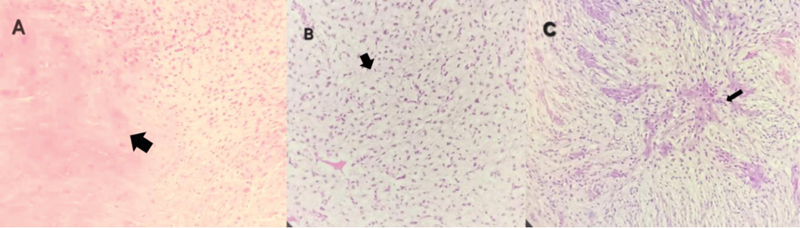
( **A** ) Moderately cellular tumor composed of spindled cells in a collagenized stroma ( *arrow* ). ( **B** ) Abrupt transition to myxoid areas ( *arrow* ). ( **C** ) Swirling growth pattern ( *arrow* ). [Hematoxylin and eosin staining (H&E), 40x].

In Fig. 2A , the tumor exhibits regions of dense, eosinophilic, collagenized stroma, containing scattered, uniform, spindle-shaped cells arranged in short fascicles. The spindle cells have elongated nuclei with fine chromatin and indistinct nucleoli. The arrow highlights an abrupt change in stromal quality transitioning toward a less collagenous area.

In Fig. 2B , a more myxoid zone is evident, characterized by a pale, loose matrix with widely spaced spindle cells. The arrow indicates the reduced cellular density and the prominence of the myxoid background, consistent with the alternating stromal pattern typical of this tumor.

In Fig. 2C , focal whorling of the spindle cells is present, forming a swirling, concentric arrangement around central hyalinized collagen. The arrow identifies a poorly formed collagen rosette, composed of central collagen deposition surrounded by radially oriented spindle cells.

Immunohistochemical (IHC) staining of the tumor cells revealed positive vimentin and weakly positive BCL2. Tumor cells tested negative for desmin, ALK, C-Kit, DOG1, Kit, CD34, EMA, LCA, MPO, β-catenin, Melan A, and HMB45. These critical findings strongly suggest a diagnosis of LGFMS in the stomach.

Molecular analysis performed at JRMS using RT-PCR identified a FUS-CREB3L1 fusion, confirming the diagnosis of LGFMS. Given the tumor's size and extent, only an incomplete laparoscopic resection was possible. Following surgery, the patient was referred to a radiation oncologist with expertise in adjuvant radiotherapy to evaluate strategies for managing the residual disease.

Consequently, she was started on doxorubicin and ifosfamide for six cycles, then switched to eribulin, with a good clinical response, and followed up to evaluate disease progression. Regular imaging studies and clinical evaluations were scheduled to ensure comprehensive surveillance.

## Discussion

Low-grade fibromyxoid sarcoma (LGFMS) is an uncommon soft tissue sarcoma defined by its misleadingly benign histological characteristics. It was first identified by Evans in 1987. 9 LGFMS usually manifests as a slow-growing, painless mass that originates in deep soft tissues of the trunk and lower extremities. 2 It is often misdiagnosed as a benign soft tissue lesion, leading to delays in appropriate management and distant metastasis. The first reported case of gastric LGFMS occurred in a Japanese female patient. 10

We reported an LGFMS characterized by standard histological findings that include fibrous and myxoid areas. The tumor cells consist of spindle cells with collagen deposition and collagen-centered clusters. Immunohistochemical analysis revealed positive staining for vimentin and BCL2 in this tumor.

The differential diagnosis for gastric LGFMS includes various soft tissue tumors such as gastrointestinal stromal tumors (GIST), schwannoma, inflammatory myofibroblastic tumors, solitary fibrous tumors, desmoid-type fibromatosis, and inflammatory fibroid polyps. It is crucial to distinguish LGFMS from these tumors, as the management and prognosis can differ significantly. Immunohistochemical and genetic tests are essential for the accurate diagnosis of LGFMS.

IHC testing showed negative KIT, CD34, and DOG1 results, indicating that this tumor is not a GIST. The absence of harmful mutations in the PDGFRA and c-KIT genes supports this conclusion.

Furthermore, negative results for genetic mutations and IHC tests for CD34, the PDGFRA gene, and β-catenin exclude inflammatory fibroid polyps and desmoid-type fibromatosis, respectively. The diagnosis of an inflammatory myofibroblastic tumor was ruled out due to the absence of inflammatory changes and a negative ALK stain in IHC analysis. Additionally, a schwannoma diagnosis was excluded because it tested negative for S100P.

In our case, we observed strong positivity for vimentin and BCL2, with microscopic examination strongly suggesting LGFS. Additionally, there was a positive result for the FUS-CREB3L1 fusion in molecular testing to support the diagnosis of LGFS.

## Conclusion

LGFMS is a rare soft tissue tumor with benign histological features, but it has a potential for local recurrence and distant metastasis. Its occurrence in the stomach is exceedingly uncommon, with only one previously reported case in the English literature. This case focuses on the importance of maintaining a high index of suspicion when encountering atypical gastric masses, especially in young adults presenting with nonspecific GI symptoms. Proper diagnosis depends on the combination of histopathological evaluation, immunohistochemical profiling, and molecular testing for specific gene fusions such as FUS-CREB3L1. Given its potential for recurrence and metastasis, a multidisciplinary approach involving surgery, oncology, radiology, and pathology is essential for optimal patient management and long-term follow-up. Our report contributes to the limited body of literature on gastric LGFMS and highlights the need for heightened awareness of this rare entity in both clinical and diagnostic settings.
